# Ursolic acid induces apoptosis and anoikis in colorectal carcinoma RKO cells

**DOI:** 10.1186/s12906-021-03232-2

**Published:** 2021-02-06

**Authors:** Jia-Lu Zheng, Shuang-Shuang Wang, Ke-Ping Shen, Lei Chen, Xiao Peng, Jin-Fang Chen, Hong-Mei An, Bing Hu

**Affiliations:** 1 Institute of Traditional Chinese Medicine in Oncology, Longhua Hospital, Shanghai University of Traditional Chinese Medicine, Shanghai, 200032 People’s Republic of China; 2 Department of Oncology, Longhua Hospital, Shanghai University of Traditional Chinese Medicine, Shanghai, 200032 People’s Republic of China; 3 Shanghai University of Traditional Chinese Medicine, Shanghai, 201203 People’s Republic of China; 4 Department of Science & Technology, Longhua Hospital, Shanghai University of Traditional Chinese Medicine, Shanghai, 200032 People’s Republic of China

**Keywords:** Ursolic acid, Colorectal cancer, Apoptosis, Caspases, Reactive oxygen species, Anoikis, FAK, PI3K, AKT, Epithelial-mesenchymal transition

## Abstract

**Background:**

Ursolic acid (UA) is an anti-cancer herbal compound. In the present study, we observed the effects of UA on anchorage-dependent and -independent growth of human colorectal cancer (CRC) RKO cells.

**Methods:**

RKO cells were cultured in conventional and detached condition and treated with UA. Cell viability was evaluated by CCK-8 assay. Cell cycle was analyzed by flow cytometry. Apoptosis was identified by Hoechst 33258 staining and flow cytometry analysis. Activities of caspases were measured by commercial kits. Reactive oxygen species (ROS) was recognized by DCFH-DA fluorescent staining. Anoikis was identified by EthD-1 fluorescent staining and flow cytometry analysis. Expression and phosphorylation of proteins were analyzed by western blot.

**Results:**

UA inhibited RKO cell viability in both a dose- and time-dependent manner. UA arrested the cell cycle at the G0/G1 phase, and induced caspase-dependent apoptosis. UA inhibited Bcl-2 expression and increased Bax expression. In addition, UA up-regulated the level of ROS that contributed to UA activated caspase-3, − 8 and − 9, and induced apoptosis. Furthermore, UA inhibited cell growth in a detached condition and induced anoikis in RKO cells that was accompanied by dampened phosphorylation of FAK, PI3K and AKT. UA also inhibited epithelial-mesenchymal transition (EMT) as indicated by the down-regulation of N-Cad expression and up-regulation of E-Cad expression.

**Conclusions:**

UA induced caspase-dependent apoptosis, and FAK/PI3K/AKT singling and EMT related anoikis in RKO cells. UA was an effective anti-cancer compound against both anchorage-dependent and -independent growth of RKO cells.

**Supplementary Information:**

The online version contains supplementary material available at 10.1186/s12906-021-03232-2.

## Background

Colorectal cancer (CRC) has the third highest malignancies incidence (10.2% of the total cases), and second highest mortality rate (9.2% of the total cancer deaths) globally [1]. It was reported that there were 1.8 million newly diagnosed CRCs, and 881,000 people died from CRC in 2018 [1]. CRC can be treated with surgery, chemotherapy and specific targeted therapies. Currently, the 5-year survival rate of locally advanced CRC can attain rates as high as 69%; however, the 5-year survival rate of metastatic CRC is only about 12% [2]. Targeted therapy includes anti-body-mediated therapy against EGFR and VEGF, and small-molecule compounds against protein kinases, such as Regorafenib. Nevertheless, the median progression-free survival (mPFS) of metastatic CRC is only 11 months, even when treated by targeted therapy combined with chemotherapy [3]. It is necessary to develop new treatment for CRC.

**Fig. 1:**
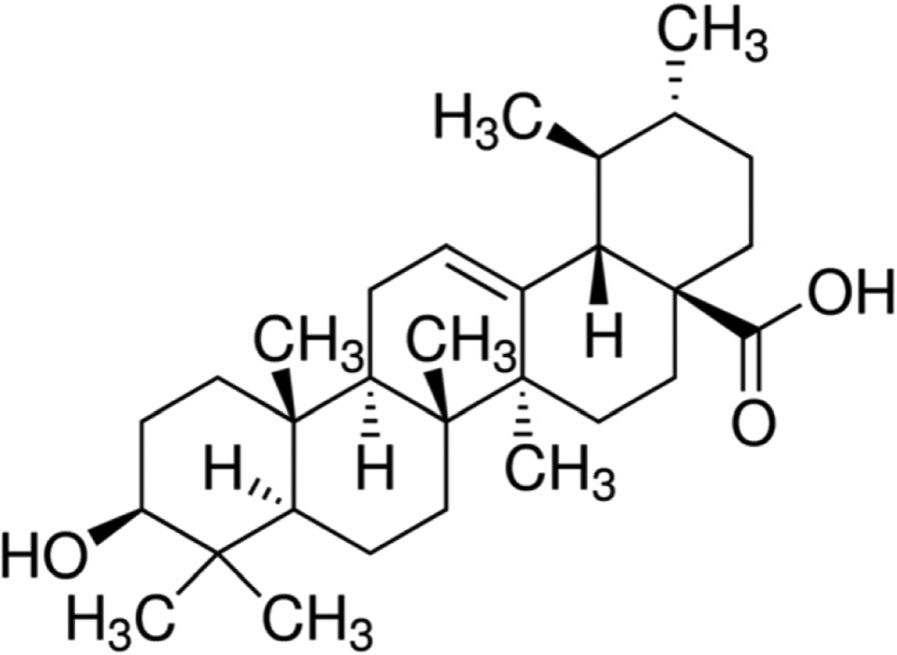
The chemical structure of UA

The dried root of *Actinidia chinensis* Planch (ACP) (Teng-Li-Gen) is a common anti-cancer traditional Chinese medicine for CRC treatment. It has been confirmed that ACP inhibits CRC cell proliferation [4]. We have demonstrated that ACP induces anoikis accompanied by reactive oxygen species (ROS) generation and caspase-3 activation in CRC RKO cells [5]. Anoikis is a type of apoptosis that occurs in epithelial cells once they have detached from the extracellular matrix (ECM) and is closely associated with cell suspension growth and tumor metastasis [6]. Ursolic acid (UA) is the major anti-cancer compound found in ACP [7] (Fig. 1). In the present study, we further observed the effects of UA on the adhesion and suspension growth of CRC RKO cells.

## Methods

### Chemicals and reagents

UA, E-Cadherin (E-Cad) and N-Cadherin (N-Cad) antibodies were obtained from Santa Cruz Biotechnology (Santa Cruz, CA, USA). Poly (2-hydroxypropyl methacrylate) (poly-HEMA) was the products of Sigma-Aldrich (St Louis, MO, USA). Trypsin-EDTA (0.25%), fetal bovine serum (FBS), Penicillin-Streptomycin (10,000 U/mL), and DMEM culture medium were obtained from Gibco (Grand Island, NY, USA). CytoSelect™ 24-Well Anoikis Assay Kit was obtained from Cell Biolabs (San Diego, CA, USA). N-acetyl-L-cysteine (NAC), Hoechst 33258 stain kit, Bradford Protein Assay Kit, Caspase activity assay kits, ROS assay kit, and Z-VAD-FMK were all the products of Beyotime (Haimen, Jiangsu, China). Cell counting kit-8 (CCK-8) 8 was from Dojindo (Shanghai, China). The Cell Cycle Kit and the FITC Annexin V Apoptosis Detection Kit were both manufactured by BD Pharmingen™ (San Diego, CA, USA). Antibodies against AKT, p-AKT, B-cell CLL/lymphoma 2 (Bcl-2), Bcl2-associated X protein (Bax), focal adhesion kinase (FAK), phosphorylated FAK (p-FAK), glyceraldehyde-3-phosphate dehydrogenase (GADPH), phosphatidylinositol 3-kinase (PI3K), and p-PI3K were all purchased from Bioworld Technology (St. Louis Park, MN, USA).

### Cell culture

Human CRC RKO cells were from the Cell Bank of Type Culture Collection of the Chinese Academy of Sciences (Cat No. TCHu116), cultured in DMEM medium (10% FBS and 1% streptomycin-penicillin), and maintained in an incubator with 5% CO_2_ and saturated humidity environment at 37 °C. RKO cells at logarithmic growth phase were used for subsequent experiments.

### Cell viability assay

A total of 1 × 10^4^ RKO cells were seeded into a conventional or poly-HEMA coated 96-well plate and cultured for 24 h, and then treated with different doses of UA or same volume of dimethyl sulfoxide (DMSO) for 48 h. Next, 10 μl of CCK-8 reagent was added to the wells, and incubated for 2 h at 37 °C. The OD450 of each well was detected using a microplate reader. The cell survival rate was calculated as follow: survival rate (%) = [experimental OD value / control OD value] × 100%.

### Flow cytometry assay

A total of 2.5 × 10^5^ RKO cells were seeded into a 6-well plate. After culturing for 24 h, the cells were treated with different doses of UA or same volume of DMSO for 48 h. The cells were collected, fixed with 75% ethanol, stained with propidium iodide (PI), and analyzed by flow cytometry for cell cycle identification. For apoptosis detection, UA treated RKO cells were sequentially stained with FITC Annexin V and PI, and then detected by flow cytometry.

### Hoechst 33258 staining

A total of 2.5 × 10^5^ RKO cells were seeded into a 6-well plate and cultured for 24 h, at which time, different concentrations of UA or an equal volume of DMSO was added. After 48 h of culture, cells were fixed, followed by staining with Hoechst33258. Cell morphology was then examined by fluorescence microscopy.

### Caspases activity detection

A total of 2.5 × 10^5^ RKO cells were seeded into a 6-well plate and cultured for 24 h, after which, the cells were treated with different concentrations of UA or same volume of DMSO for 48 h, and harvested. Activities of caspases were detected by commercial kits in which Ac-DEVD-pNA, Ac-IETD-pNA and Ac-LEHD-pNA were used as the substrates of caspases-3, − 8 and − 9, respectively. To address the role of caspases in UA-induced apoptosis, RKO cells were pre-treated with 100 μM of Z-VAD-FMK (a caspases inhibitor) for 2 h, followed by UA treatment and apoptosis detection.

### Quantification of ROS

ROS was measured according to the manufacturer’s protocol. Briefly, 2.5 × 10^5^ RKO cells were seeded into a 6-well plate, cultured for 24 h, and treated with different doses of UA or an equal volume of DMSO for 48 h. The cells were then stained with 2′,7′-Dichlorofluorescin diacetate (DCFH-DA) (diluted at 1:1000) for 20 min, observed by fluorescence microscopy, and detected by a fluorescent plate reader (excitation wavelength of 488 nm and an emission wavelength of 525 nm). For inhibition of ROS, RKO cells were pre-incubated with NAC (200 μM) for 2 h, followed by treatment with UA, and subsequent detection of caspases and apoptosis.

### Anoikis detection

A total of 2.5 × 10^5^ RKO cells were seeded into a poly-HEMA coated 24-well plate and cultured for 24 h. Next day, the cells were treated with different doses of UA or same volume of DMSO for 48 h, and stained with 1 μl of EthD-1 for 1 h at 37 °C. The fluorescence was observed under a microscope, and measured by a fluorescence microplate reader (set at an excitation wavelength of 525 nm and an emission wavelength of 590 nm). For identification of apoptosis, the UA treated RKO cells that were grown in suspension, were sequentially stained with FITC Annexin V and PI, and then analyzed by flow cytometry.

### Western blotting

Protein expression and phosphorylation was evaluated by Western blotting as described previously [8, 9]. Briefly, UA treated RKO cells were harvested, lysed and quantified. Proteins were separated by sodium dodecyl sulfate-polyacrylamide gel electrophoresis (SDS-PAGE) (8 – 10%), and transferred to a polyvinylidene fluoride (PVDF) membrane under semi-dry condition. After blocked with 5% non-fat milk, the membranes were incubated with antibodies against Bcl-2 and Bax (at a dilution of 1:1000), AKT, p-AKT, FAK, p-FAK, PI3K and p-PI3K (at a dilution of 1:700), E-Cad and N-Cad (at a dilution of 1:500) or GAPDH (at a dilution of 1:2000) at 4 °C overnight, and washed with PBST. The blots were probed with horseradish peroxidase (HRP)-conjugated secondary antibody (1:2000) for 1 h, washed in PBST, and developed by enhanced chemiluminescence (ECL) reagent.

### Statistical analysis

The data were expressed as mean ± standard deviation (SD). The differences between groups were compared by One-way analysis of variance (ANOVA), and statistically differences were considered at *p <* 0.05.

## Results

### UA inhibits cell viability

**Fig. 2:**
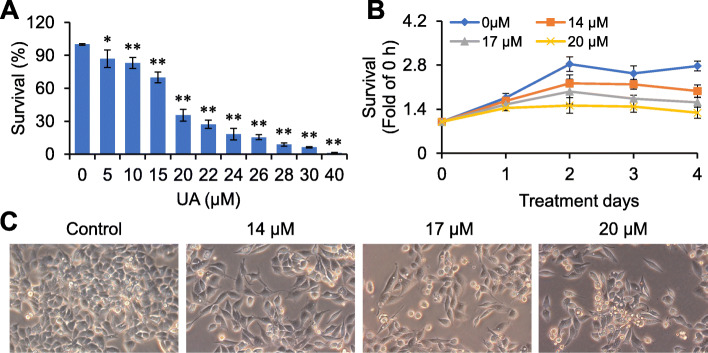
Effects of UA on RKO cells growth. **a** and **b**, RKO cells were dose-and time-dependently treated with UA, and cell vitality were evaluated by CCK-8 assay. **c**, RKO cells were treated with UA (14–20 μM) for 48 h, and observed by microscopy (× 200 magnification). ^*^p < 0.05, ^**^p < 0.01, versus control group

We first observed the effects of UA on RKO cells viability. As shown in Fig. 2a, UA significantly inhibited the viability of RKO cells in a dose-dependent manner (*p <* 0.05), the half maximal inhibitory concentration (IC50) value of UA was 18.54 μM. RKO cells growth was completely inhibited by 40 μM of UA. Thus, 14–17 μM UA was selected for subsequent studies. Further study showed that 14–17 μM UA significantly inhibited RKO cells viability in a dose- and time-dependent manner (*p <* 0.01) (Fig. 2b). Meanwhile, the distribution density of RKO cells was decreased after UA treatment (Fig. 2c).

### UA induces cell cycle arrest

**Fig. 3:**
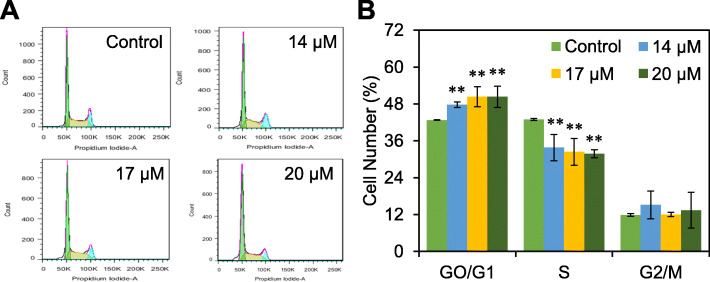
UA arrests cell cycle. RKO cells were treated with UA (14–20 μM) for 48 h and cell cycle distribution were analyzed by flow cytometry (**a**) and the results were expressed as mean ± SD (**b**). ^**^p < 0.01, versus control group

The effects of UA on cell cycle were analyzed by PI labeling and flow cytometry. The results were shown in Fig. 3, RKO cells at the G0/G1 phase increased (*p <* 0.01), while cells at the S phase decreased correspondingly (*p <* 0.01) after treating with 14–17 μM UA for 48 h. These observations suggested that UA can arrest RKO cell cycle at the G0/G1 phase.

### UA induces apoptosis

**Fig. 4:**
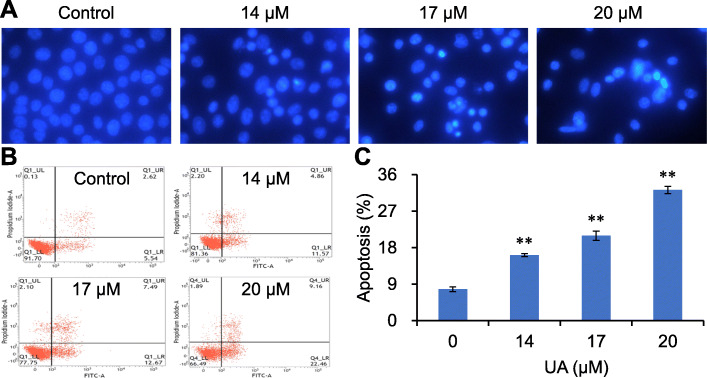
UA induces apoptosis. UA treated RKO cells were subjected to Hoechst 33258 staining (**a**), flow cytometry analysis for apoptosis detection (**b**) and expressed by mean ± SD (**c**). ^**^p < 0.01, versus control group

Apoptosis is an important mechanism for natural products to treat cancer [10]. In our study, Hoechst 33258 staining revealed that the distribution density of RKO cells decreased after treating with 14–17 μM UA for 48 h. Moreover, some cells were densely stained bright blue and exhibited nuclear fragmentation (Fig. 4a), suggesting the occurrence of apoptosis. In addition, FITC Annexin V/PI double staining showed that 14–17 μM UA promoted apoptosis of RKO cells (*p <* 0.01) (Fig. 4b and c).

### UA activates Caspases

**Fig. 5:**
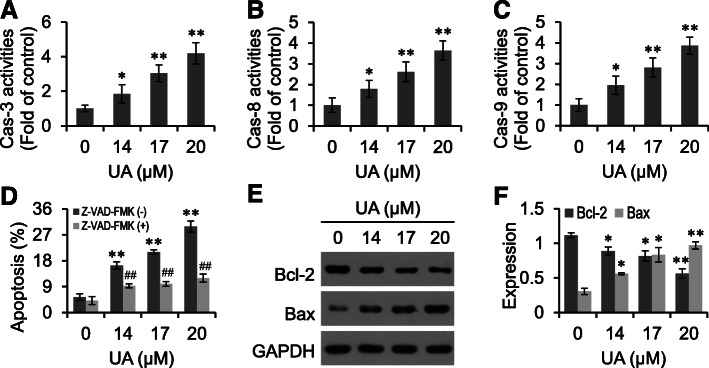
UA activates Caspases. Caspase-3 (**a**), − 8 (**b**) and − 9 (**c**) activities in UA treated RKO cells. **d**, After pre-incubated with Z-VAD-FMK for 2 h, RKO cells were treated with UA and subjected to apoptosis detection. Bcl-2 and Bax expression in UA treated RKO cells were evaluated by Western blot (**e**), and analyzed by the Quantity One software (**f**). ^*^p < 0.05, ^**^p < 0.01, versus control group; ^#^*p <* 0.05, ^##^*p <* 0.01, versus UA group

Apoptosis is the caspase cascade-mediated cell death, which is closely correlated with the activation of caspases [10, 11]. Results of our study revealed that 14–17 μM UA significantly activated caspase-3, − 8 and − 9 in RKO cells (*p <* 0.05) (Fig. 5a-c). In addition, caspases inhibitor Z-VAD-FMK significantly antagonized UA induced apoptosis (*p <* 0.01) (Fig. 5d). These results suggest that caspases were involved in UA-induced apoptosis of RKO cells. Moreover, UA inhibited Bcl-2 expression and up-regulated Bax expression (*p <* 0.05) (Fig. 5e and f, Supplementary Fig. 1).

### UA increases ROS

**Fig. 6:**
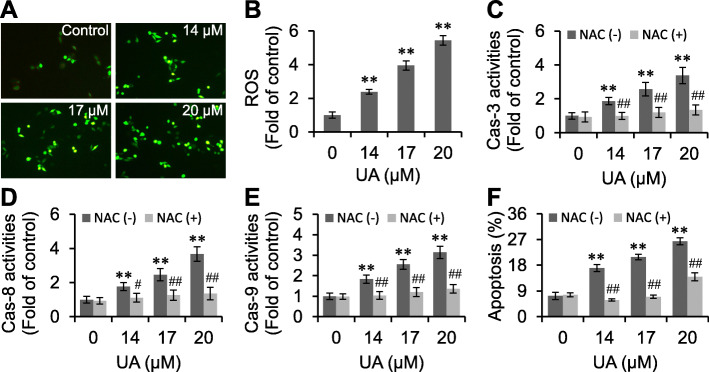
UA increases ROS. RKO cells were treated with UA, stained with DCFH-DA, observed under a microscope (× 200) (**a**), and the fluorescence were detected by plate reader (**b**). After pre-incubated with NAC for 2 h, RKO cells were treated with UA, and subjected to detection of Caspase-3 (**c**), − 8 (**d**) and − 9 (**e**) activities and apoptosis (**f**). ^**^p < 0.01, versus control group; ^#^*p <* 0.05, ^##^*p <* 0.01, versus UA group

A high level of ROS induces cellular apoptosis [12]. In this study, DCFH-DA staining showed that 14–17 μM UA significantly increased the ROS levels in RKO cells (*p <* 0.01) (Fig. 6a and b). In the meantime, NAC, a ROS scavenger, could counteract UA increased activities of caspase-3, − 8 and − 9, and promoted apoptosis (*p <* 0.01) (Fig. 6c-f). These results demonstrated that ROS participated in the activation of caspases and induction of apoptosis elicited by UA.

### UA induces anoikis

**Fig. 7:**
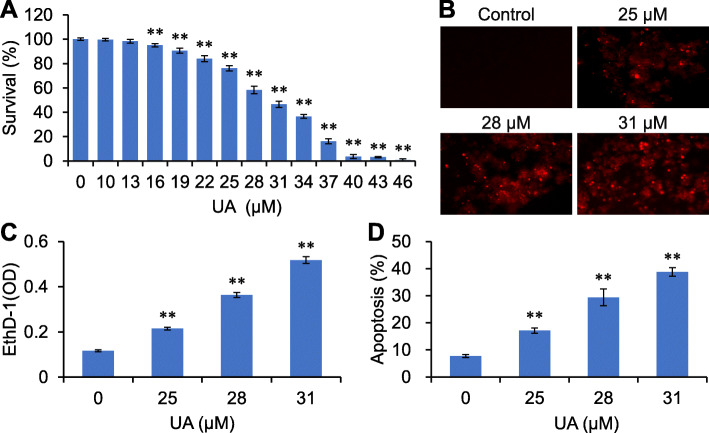
UA induces anoikis. RKO cells grown in ploy-HEMA coated plates were treated with UA for 48 h and subjected to cell viability assay (**a**), EthD-1 staining (× 200) (**b**), and the red fluorescence detected by plate reader (**c**), stained with FITC Annexin V and PI and analyzed by flow cytometry (**d**). ^**^p < 0.01, versus control group

We have observed that ACP induces anoikis in RKO cells [5]. UA, a major component in Teng-Li-Gen, may have similar effects on RKO cells. In the present study, poly-HEMA were used to reduce cell adhesion. As shown in Fig. 7a, UA inhibited RKO cells growth in a detached condition, the IC50 value of UA was 31.81 μM. Growth of suspended RKO cells was completely inhibited by 46 μM UA (*p <* 0.01). In addition, the anoikis assay showed that suspension grown RKO cells could absorb EthD-1 and emit red fluorescence following UA treatment (*p <* 0.01) (Fig. 7b and c), which indicated the occurrence of anoikis. Moreover, FITC Annexin V/PI double staining showed that UA promoted apoptosis in suspension grown RKO cells (*p <* 0.01) – which we believe to be anoikis (Fig. 7d).

### UA inhibits FAK signaling

**Fig. 8:**
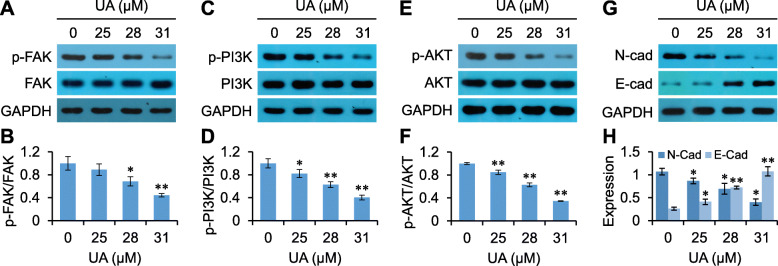
UA regulates anoikis-related proteins. Proteins expression and phosphorylation in UA treated RKO cells were evaluated by Western blot with indicated antibodies (**a**, **c**, **e** and **g**), and analyzed by the Quantity One software (**b**, **d**, **f** and **h**). ^*^p < 0.05, ^**^p < 0.01, versus control group

FAK is an important protein regulating anoikis, and its downstream signaling is closely related to the PI3K/AKT pathway [13]. In the present study, the expression and phosphorylation of these proteins was identified by Western blotting. Results demonstrated that UA did not significant affect FAK, PI3K or AKT expression in suspension grown RKO cells. However, UA inhibited the phosphorylation levels of these proteins in suspension grown RKO cells in a dose-dependent manner (Fig. 8a-f, Supplementary Fig. 2A-I).

### UA inhibits epithelial-mesenchymal transition (EMT)

Anoikis resistance is closely related to EMT [13, 14]. UA has demonstrated inhibitory effects on EMT in virous cancer cells, including CRC cells [15–17]. In the present study, the expression of EMT marker proteins were detected by Western blotting. The results showed that UA inhibited N-Cad expression (mesenchymal marker) and up-regulated E-Cad expression (epithelial marker) (Fig. 8g and h, Supplementary Fig. 2 J-L), suggesting that UA inhibited EMT in RKO cells.

## Discussion

Cancer cells proliferate in an uncontrolled manner, for this reason, inhibiting cell proliferation is the basic principle of cancer therapy [18]. Results of this current study showed that UA significantly inhibited RKO cells growth. Meanwhile, cell cycle analysis demonstrated that RKO cells at the G0/GI stage increased, while cells at the S phase decreased after UA treatment. Since cell proliferation is a self-perpetuating process, at least in part, on the basis of cell cycle kinetics, it suggests that cell cycle arrest was involved in blocking RKO cell growth by UA treatment. Furthermore, Hoechst 33258 and Annexin V/PI staining revealed that UA also promoted apoptosis of RKO cells.

Apoptosis is regulated by a cascade of protein-mediated reactions, which is an important mechanism of cancer treatment [19]. Apoptotic cells display characteristic and specific morphologies, including apoptotic bodies and DNA fragmentation. The apoptotic process is mainly associated with mitochondrial/intrinsic and death receptor/extrinsic pathways, and regulated by multiple proteins, such as Bcl-2 and Bax. In the mitochondrial pathway, cytotoxic signals decrease the mitochondrial membrane potential (MMP) and elicit the release of cytochrome C, thus activating caspase-9 and -3 to initiate apoptosis. In the death receptor pathway, death ligands, such as FasL, TNF and TRAIL, associate with their corresponding receptors and sequentially activate caspase-8 and -3 to provoke apoptosis. The present study showed that UA activated caspases-8, − 9 and − 3. Meanwhile, Z-VAD-FMK antagonized UA induced apoptosis, suggesting that caspases were involved in UA-induced apoptosis.

Apoptosis is associated with a variety of factors, including ROS production [12, 20]. ROS are intracellularly active oxygen species, which include. OH, O_2_•^−^ and H_2_O_2_. High level of ROS induces apoptosis by activating caspase-8 or caspase-9 via the mitochondrial or death receptor pathways. DCFH-DA can freely penetrate the cell membrane, which can be hydrolyzed to form DCFH by the esterase, thus generating 2′,7′-dichlorofluorescein (DCF) with fluorescence intensity that is associated with the effects of ROS. The fluorescence intensity of DCF reflects the relative levels of intracellular ROS. DCFH-DA staining showed that RKO cells emitted green florescence after UA treatment, indicating that UA increased ROS levels. Further study showed that NAC antagonized UA activated caspase-3, − 8 and − 9 and promoted apoptosis. These observations suggested that ROS was involved in the apoptosis-inducing effects of UA in RKO cells.

We further observed the effects of UA on suspension growth of RKO cells by using poly-HEMA to reduce cell adhesion. The results showed that UA inhibited the suspension growth and stimulated anoikis in RKO cells. Anoikis is a type of apoptosis occurred in epithelial cells due to the detachment from ECM, including epithelial cancer cells, and thus to be known as detachment-induced apoptosis [21]. FAK is an important protein regulating anoikis [13]. FAK activation provides survival signals to prevent anoikis, and does so via the PI3K/AKT signaling pathway. Inhibiting FAK can stimulate anoikis [22]. Results from the present study demonstrated that UA inhibited phosphorylation of FAK, PI3K and AKT in suspension grown RKO cells, suggesting that UA-induced anoikis might be associated with these proteins.

EMT is related to anoikis resistance [13, 14]. EMT is the process by which epithelial cells, including epithelial cancer cells, lose epithelial characteristics and gain mesenchymal phenotype, accompanied by reduced epithelial marker E-Cad expression and increased mesenchymal marker N-Cad expression. PI3K/AKT signal transduction contributes to EMT [23, 24]. The present study showed that UA could increase E-Cad expression, reduce N-Cad expression, and accompanied by down-regulation of PI3K/AKT singling, suggesting that UA could inhibit EMT and may related to PI3K/AKT singling.

## Conclusions

In summary, the present study suggested that UA inhibits cell viability, induces cell cycle arrest at the G0/G1 phase, and regulates Bcl-2/Bax expression, increases ROS leading to activation of caspase-3, − 8 and − 9, as well as inducing apoptosis in RKO cells. UA also inhibits suspension growth of RKO cells, elicits anoikis, and may related to FAK/PI3K/AKT singling and inhibition of EMT. This study provided the fundamental basis for applying UA to treat colorectal cancer.

## Supplementary Information

**Additional file 1: Supplementary Figure 1**. UA regulates Bcl-2 and Bax expression. UA treated RKO cells were subjected to Western blot with antibodies against Bcl-2 (A), Bax (B), and GAPDH (C). **Supplementary Figure 2**. UA regulates anokis related proteins expression. UA treated RKO cells were subjected to Western blot with antibodies against p-FAK (A), FAK (B), p-PI3K (D), PI3K (E), p-AKT (G), AKT (H), N-Cad (J), E-Cad (K), and GAPDH (C, F, I and L).

## Data Availability

The datasets used and/or analyzed during the current study available from the corresponding author on reasonable request.

## Abbreviations

CCK-8: Cell counting kit-8
CRC: Colorectal cancer
DCFH-DA: 2′,7′-Dichlorofluorescin diacetate
DMSO: Dimethyl sulfoxide
FAK: Focal adhesion kinase
GAPDH: Glyceraldehyde-3-phosphate dehydrogenase
IC50: Half maximal inhibitory concentration
NAC: N-acetyl-L-cysteine
OD: Optical density
PI: Propidium iodide
PI3K: Phosphatidylinositol 3-kinase
poly-HEMA: Poly (2-hydroxyethyl methacrylate)
ROS: Reactive oxygen species
SD: Standard deviation
UA: Ursolic acid
