# Supplementary material for: Ursolic acid induces apoptosis and anoikis in colorectal carcinoma RKO cells
Source: BMC Complement Med Ther. 2021 Feb 6;21:52. doi: 10.1186/s12906-021-03232-2 (PMC7866452; doi:10.1186/s12906-021-03232-2)
Supplement: Supplementary file 1 — Additional file 1: Supplementary Figure 1. UA regulates Bcl-2 and Bax expression. UA treated RKO cells were subjected to Western blot with antibodies against Bcl-2 (A), Bax (B), and GAPDH (C). Supplementary Figure 2. UA regulates anokis related proteins expression. UA treated RKO cells were subjected to Western blot with antibodies against p-FAK (A), FAK (B), p-PI3K (D), PI3K (E), p-AKT (G), AKT (H), N-Cad (J), E-Cad (K), and GAPDH (C, F, I and L). [file 12906_2021_3232_MOESM1_ESM.docx]

Supplementary Figure 1. UA regulates Bcl-2 and Bax expression. UA treated RKO cells were subjected to Western blot with antibodies against Bcl-2 (A), Bax (B), and GAPDH (C).

Supplementary Figure 2. UA regulates anokis related proteins expression. UA treated RKO cells were subjected to Western blot with antibodies against p-FAK (A), FAK (B), p-PI3K (D), PI3K (E), p-AKT (G), AKT (H), N-Cad (J), E-Cad (K), and GAPDH (C, F, I and L).
